# Multi-agent system collision model to predict the transmission of seasonal influenza in Tokyo from 2014–2015 to 2018–2019 seasons

**DOI:** 10.1016/j.heliyon.2021.e07859

**Published:** 2021-08-23

**Authors:** Nobuo Tomizawa, Kanako K. Kumamaru, Koh Okamoto, Shigeki Aoki

**Affiliations:** a Department of Radiology, Juntendo University Graduate School of Medicine, Tokyo, Japan; b Department of Infectious Diseases, The University of Tokyo Hospital, Tokyo, Japan

**Keywords:** Disease transmission, Influenza, Multi-agent system, SIR model

## Abstract

The objective of this study was to apply the multi-agent system (MAS) collision model to predict seasonal influenza epidemic in Tokyo for 5 seasons (2014–2015 to 2018–2019 seasons). The MAS collision model assumes each individual as a particle inside a square domain. The particles move within the domain and disease transmission occurs in a certain probability when an infected particle collides a susceptible particle. The probability was determined based on the basic reproduction number calculated using the actual data. The simulation started with 1 infected particle and 999 susceptible particles to correspond to the onset of an influenza epidemic. We performed the simulation for 150 days and the calculation was repeated 500 times for each season. To improve the accuracy of the prediction, we selected simulations which have similar incidence number to the actual data in specific weeks. Analysis including all simulations corresponded good to the actual data in 2014–2015 and 2015–2016 seasons. However, the model failed to predict the sharp peak incidence after the New Year Holidays in 2016–2017, 2017–2018, and 2018–2019 seasons. A model which included simulations selected by the week of peak incidence predicted the week and number of peak incidence better than a model including all simulations in all seasons. The reproduction number was also similar to the actual data in this model. In conclusion, the MAS collision model predicted the epidemic curve with good accuracy by selecting the simulations using the actual data without changing the initial parameters such as the basic reproduction number and infection time.

## 1 Introduction

Seasonal influenza epidemics result in nearly 3 to 5 million cases of severe illness a year and have a great importance in public healthcare [1]. Influenza also causes cardiovascular disorders as well as other complications [2]. Therefore, controlling and preventing the epidemic of influenza is an important issue [3]. Mathematical models, such as truncated model and the SIR model [4, 5], have been introduced to predict the transmission of infectious diseases [6]. The strength of these models is the simplicity of calculation due to the deterministic nature and the results would be identical for fixed initial values. However, disease transmission is a sum of many small individual effects, and random events cannot be ignored [7]. Therefore, stochastic models might predict disease transmission better than deterministic models.

A multi-agent system (MAS) model is a stochastic method to predict various phenomena. MAS approach has been applied for hepatitis C virus infection modelling [8], pre-hospital emergency management [9], real-time scheduling for out-patient clinics [10], tumor growth [11], and immune responses [12]. Stochastic spatial models have also been applied to epidemic forecasting [13, 14]. Most of these studies apply a mathematical method to estimate the interaction between different compartments. In contrast, by representing each individual as a particle, collision of particles would correspond to interaction between individuals. A previous study introduced a kinetic model of mobile susceptible and infective individuals in a two-dimensional domain [15]. They applied this model to predict the epidemic curve of measles. However, this was an *in vitro* study which compared to the SIR model. We referred to this model as a MAS collision model and sought that this model could be applied for prediction of actual seasonal influenza epidemic. Recent studies using MAS models attempt to increase the precision by including multiple parameters in order to simulate the daily schedule of each individual [16, 17]. However, the calculation cost increase tremendously when sophisticated models are used, and a supercomputer would be necessary to perform these methods. Conversely, a simple model just focusing on collision might be able to predict the influenza epidemic using a commercially available computer. Our hypothesis was that a simplified model focusing only on collision of people with calibration using data of the first 4 weeks after onset could predict the epidemic curve of seasonal influenza. Therefore, the purpose of this study was to apply the MAS collision model to predict seasonal influenza epidemic in Tokyo for 5 seasons.

## 2 Methods

### 2.1 Data

Weekly sentinel influenza surveillance in Tokyo is performed in 419 clinics or hospitals. Weekly data of new cases per site are available at the Tokyo Metropolitan Infectious Disease Surveillance Center website (http://idsc.tokyo-eiken.go.jp/diseases/flu/flu/, Supplemental Table 1). A case is reported 1) if a patient has all four clinical symptoms (high-grade fever, malaise, cough, and sore throat of sudden onset) or 2) if a patient has some symptoms and is tested positive for influenza via a rapid antigen detection by immunochromatography using a nasopharyngeal swab sample [18]. The influenza season starts in the 36^th^ week and ends in the 35^th^ week of the next year. We collected the data of 5 seasons: from 2014-2015 to 2018–2019 seasons.

The influenza epidemic threshold was defined as weekly onset of >1 patient per site. We started the prediction model at the onset of the corresponding season. The basic reproduction number (*R*_*0*_) for each season was determined as the mean reproduction number of 5 weeks including the onset of the epidemic: for example, if the influenza epidemic started at week *N*, we calculated the mean reproduction number from week *N*−2 to *N*+2. The calculation method of the reproduction number is described below.

### 2.2 The MAS collision model

#### 2.2.1 Hardware and software

Model experiments including the MAS collision model and the SIR model were performed on a computer with 16 GB CPU memory, an Intel Core i7-7700 3.60 GHz CPU (Intel, Santa Clara, CA), using Python 3.7.

#### 2.2.2 Basic principle

**Figure 1:**
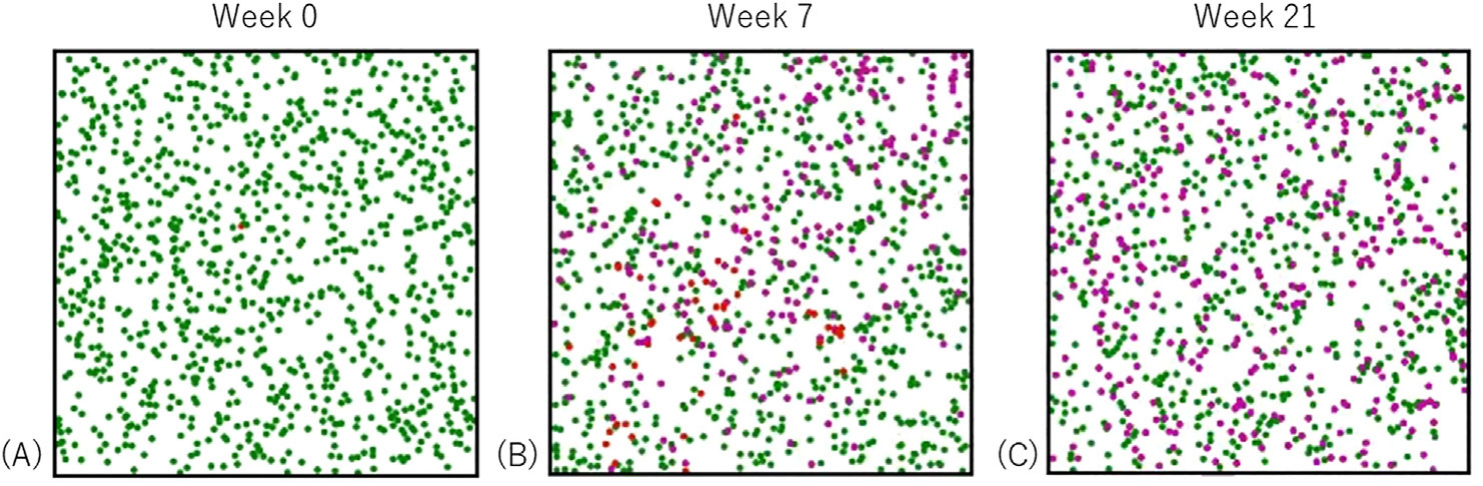
A sample image of simulation number 102 in 2018–2019 season. We assumed each individual as a particle. The particle color represents the status: green, red, and purple particles correspond to susceptible, infectious, and removed individuals, respectively. Initially, one particle is infectious while the remaining particles are susceptible (A). At week 7, 49 particles are infectious while 188 particles are removed (B). Finally, at week 21, no particles are no longer infectious, and 353 particles are removed (C). A total of 647 particles remained susceptible.

We assumed each individual as a particle inside a square domain (0 ≤ x ≤ 1, 0 ≤ y ≤ 1) with a radius of 0.0075. The initial interparticle spacing was approximately 0.03 in this simulation. Susceptible, infectious, and removed individuals were drawn as green, red, and purple particles, respectively (Figure 1). The initial position of each particle was selected randomly. To correspond to a heterogenous population, the initial particle velocity was randomly determined based on a normal distribution with a standard deviation of 0.1 times the mean speed. The particles move within the domain and are elastically reflected off the walls. When two particles collide, the velocities change such that both energy and momentum is conserved.

We determined the total number of particles as 1,000 to correspond to the number of covered people per clinic or hospital in Tokyo. The estimation was performed using the following data. First, the number of clinics and hospitals in Tokyo was 13,429 and 647, respectively, in 2018, which is available at the Bureau of Social Welfare and Public Health website (http://www.fukushihoken.metro.tokyo.lg.jp/kiban/chosa_tokei_iryosisetsu/heisei30nen.html). Next, the population of Tokyo in December 2018 was 13,859,764. This leads to a mean population coverage of 985 people per clinic or hospital.

#### 2.2.3 Particle velocity and number of collisions

Before performing the main experiment, we investigated the relationship between the mean particle velocity and the number of collisions. The number of particles and the size of the domain was the same as the main experiment. A total of 12 frames were performed in each simulation and the total number of collisions was recorded. We performed this experiment in 23 different particle velocities ranging from 0.56 to 1.00 with a step of 0.02, and 50 simulations were performed for each velocity.

#### 2.2.4 Main experiment

The initial number of susceptible and infectious individulals was 999 and 1, respectively, because epidemic onset was defined as >1 patient per clinic or hospital and each site covers a population of approximately 1,000. Based on the preliminary experiment, we adopted 0.98 as the mean initial particle velocity. This makes approximately 250 collisions per frame. We defined 6 frames to correspond to a single day. This results in 3 contacts per particle per day (250 × 6×2/1,000). Note that because 1 collision account for 1 contact with each particle, the total contacts would be the twice of the collision number. We performed the simulation for 900 frames (900/6 = 150 days).

**Figure 2:**
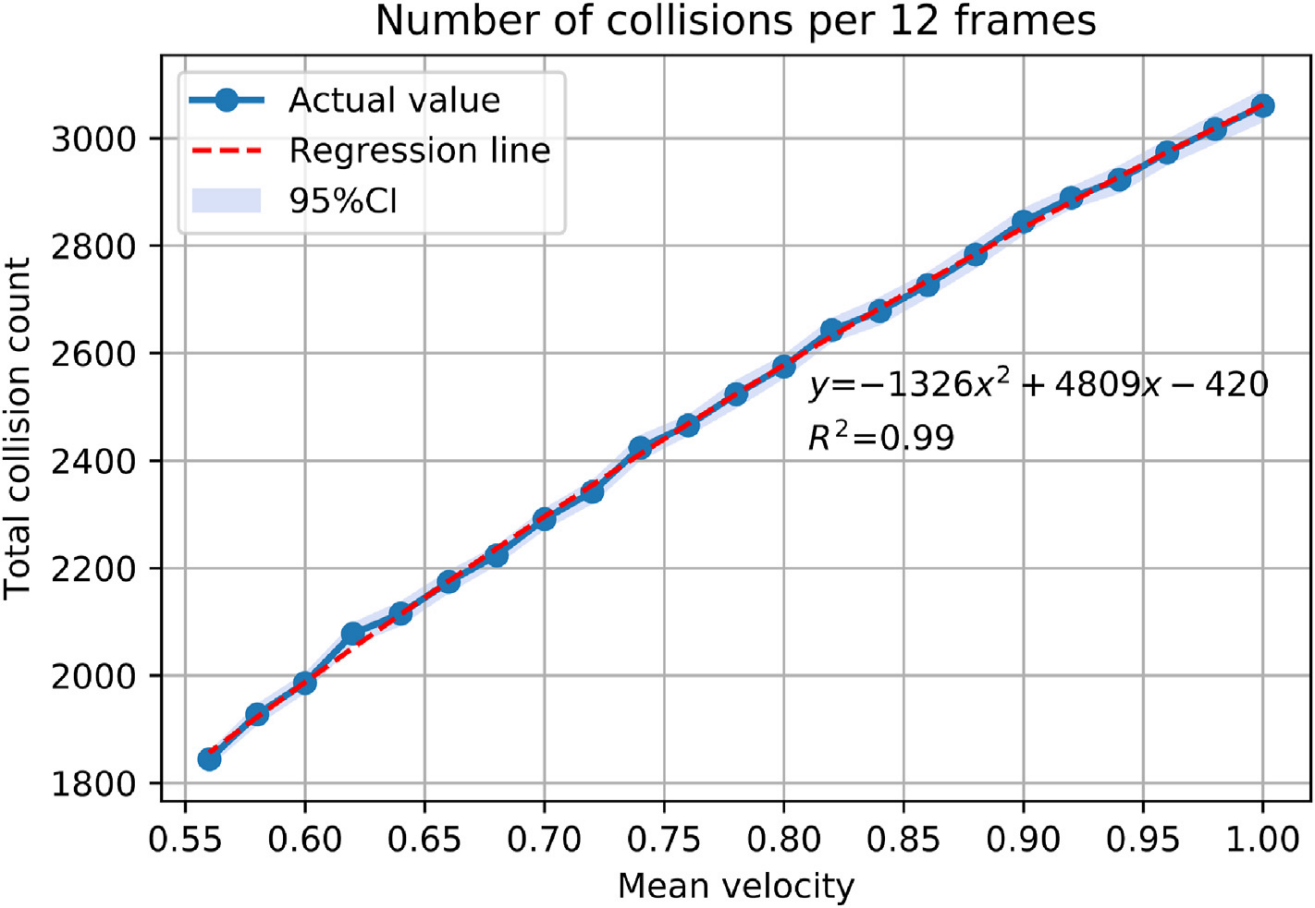
Relationship between the mean particle velocity and the number of collisions. Quadratic regression analysis (red dotted line) showed a strong positive correlation between the two values (*R*^*2*^ = 0.99). CI, confidence interval.

In Japan, the activity of people decreases during the New Year Holidays. The number of new influenza patients reduces during the holidays each year. We performed simulations using the SIR model described below with various reproduction numbers during the holidays (data not shown). We determined that reduction of reproduction number to 75% during the 51^st^ and 52^nd^ week would be feasible. This accounts for reduction in particle velocity to 70% based on the preliminary experiment (Figure 2). We did not perform velocity reduction in 2015–2016 season because the epidemic started in the 1^st^ week of 2016.

The infectious period (1/γ) was estimated as 5 days [6, 19]. Therefore, an infectious individual turns removed in 5 days (30 frames). When an infectious individual collides a susceptible individual, the susceptible individual turns infectious with a predefined probability. The probability was calculated from the *R*_*0*_ determined from the actual influenza surveillance data using the following method. An infectious individual transmits to a total of *R*_*0*_ susceptible individuals during the infectious period (1/γ). The infectious individual contacts 1 ∕ γ×3 times during the infectious period. Therefore, the probability of an infectious individual to transmit to a susceptible individual would be *R*_*0*_×γ ∕ 3 per contact. To correspond to this probability, a random number between 0 to 1 were generated when an infectious individual collides a susceptible individual, and the susceptible individual would turn infectious when the number was < *R*_*0*_×γ ∕ 3.

To explore the closeness of the estimation of the model to the actual incidence number, we performed 500 simulations for each season.

### 2.3 Comparison with the SIR model

The SIR model is a compartment model to describe the transmission of infectious disease [5]. All individuals are classified as one of the 3 compartments: susceptible (S), infected (I), and removed (R). The total number of individuals (*N* = *S*(*t*)+*I*(*t*)+*R*(*t*)) is fixed to 1,000. The model is described by the following ordinary differential equations.

$$\frac{dS}{dt}=-\frac{\beta IS}{N}$$

$$\frac{dI}{dt}=\frac{\beta IS}{N}-\gamma I$$

$$\frac{dR}{dt}=\gamma I$$

β is the transmission rate, calculated by multiplying *R*_*0*_ and γ. The β value was calculated from the *R*_*0*_ value, and we used a fixed γ value of 0.2 as described above. The initial values were determined as follows: *S*(0) = 999, *I*(0) = 1, *R*(0) = 0. We performed the simulation for 150 days.

### 2.4 Estimation of reproduction number

#### 2.4.1 Reproduction number using weekly incidence

The actual data for influenza incidence was reported weekly. We performed a following numerical analysis to estimate the reproduction number using the adjacent weekly incidence data. First, we started with 1 infected individual at day 0. When the reproduction number is 1 and the infectious period is 5 days, the individual will transmit to 0.2 individuals in days 1–5. Next, incident individuals at day 1 will transmit to 0.04 (= 0.2 ∕ 5) individuals in days 2–6. Furthermore, incident individuals at day 2 (0.24 = 0.2 + 0.04) will transmit to 0.048 (= 0.24 ∕ 5) individuals in days 3–7. Daily incidence of influenza patients could be obtained by repeating this method. Using this data, we calculated the ratio of weekly incidence which is defined as (weekly incidence at week *N*+1) ∕ (weekly incidence at week *N*). Because peak in daily incidence number is observed every 5 days during the first few weeks, the ratio will not stabilize until approximately week 7 (data not shown). Therefore, we calculated the daily incidence to 70 days and used the data of week 10 for analysis. We performed this analysis with a reproduction number between 0.30 and 2.50 with a step of 0.01 (Supplemental Table 2). We used this table to estimate the reproduction number when only weekly data is available.

#### 2.4.2 Reproduction number using daily incidence

The reproduction number *R*(*t*) can be estimated by the ratio of the number of new infections generated at time *t*, *N*(*t*), to the total incident individuals at time *t*, given by $\overset{t}{\underset{s=1}{\sum }}N\left(t-s\right)w\left(s\right)$, where *w*(*s*) is the weighting factor of the infectivity [20]. In practice, transmissibility can change over time, and the generation time distribution is difficult to measure. Given that the infectious period was set to 5 days, we estimated that *w*(*s*) = 0.2 during *s* = 1 to 5, otherwise *w*(*s*) = 0. In the real world, the viral shedding and the transmission potential is highest just after the onset and declines thereafter [19, 21]. We assumed the transmission potential as the same during the infectious period to simplify the model in this study. We further calculated the reproduction number over sliding weekly windows.

### 2.5 Statistical analysis

#### 2.5.1 The MAS collision model: preliminary experiment

A regression analysis was performed to assess the relationship between the mean particle velocity and the number of collisions. We used a quadratic regression analysis rather than a linear regression analysis because a quadratic analysis fitted better than linear analysis in low and high velocities.

#### 2.5.2 The MAS collision model: main experiment

Weekly new patients were recorded in each simulation. The means and 95% confidence intervals were calculated for each season. Because this model is a stochastic model, the number of infected patients in total varied from approximately <10 to >500 patients. In order to improve the prediction, we applied the following filter as a checkpoint at specific weeks to select the simulations for prediction analysis: when the number of weekly incidence was N(*i*) in week *i*, simulations with weekly incidence between N(*i*)×0.6 and N(*i*)×1.4 were eligible. We analyzed the simulated data using the following 4 models by applying the filter: Model 1, include all data with no exclusion; Model 2, apply the filter at week 2; Model 3, apply the filter at weeks 2 and 4; Model 4, apply the filter at the weeks with peak number of incidence before and after the New Year Holidays. Only a single peak was found in the 2015–2016 season. Therefore, we applied the filter at the week with peak incidence and 4 weeks later. We recorded the calculation time for each simulation.

#### 2.5.3 The SIR model

The ordinary differential equations were solved using the odeint function found at scipy.integrate class. We assigned the initial state (numbers of susceptible, infected, and removed individuals), time interval for calculation, and the basic parameters (β and γ) in the function. The time interval was 1/100 day for calculation. The function gives the state of each compartment (susceptible, infected, and removed individuals) by numerically solving the equation. We recorded the daily status of each compartment.

#### 2.5.4 Comparison of models

We calculated the mean absolute error (MAE), root mean squared error (RMSE), and mean absolute percentage error (MAPE) to compare the accuracy of the models. The calculations were performed as follows.

$$MAE=\frac{1}{n}\sum_{t=1}^{n}\left|P_{t}-A_{t}\right|$$

$$RMSE=\sqrt{\frac{1}{n}{\sum_{t=1}^{n}\left(P_{t}-A_{t}\right)}^{2}}$$

$$MAPE=\frac{1}{n}\sum_{t=1}^{n}\left|\frac{P_{t}-A_{t}}{A_{t}}\right|\times 100\%$$

where *P*_*t*_ is the predicted value and *A*_*t*_ is the actual value.

#### 2.5.5 Sensitivity analysis

We performed a qualitative sensitivity analysis in Model 1 to assess the robustness of the calculation [22]. The week and number of maximal weekly incidence of influenza patients was calculated using the first and last 250 simulations. We compared the results with the data using all simulations.

## 3 Results

### 3.1 Actual epidemic data

The influenza epidemic started in the 47^th^, 46^th^, 47^th^, and 49^th^ week of 2014–2015, 2016–2017, 2017–2018, and 2018–2019 season, respectively. The epidemic of 2015–2016 season started in the 1^st^ week of 2016. Therefore, decrease in the weekly incidence during the New Year Holidays was not observed in this season. The *R*_*0*_ were estimated as follows: 2014–2015 season, 1.31; 2015–2016 season, 1.32; 2016–2017 season, 1.18; 2017–2018 season, 1.21; 2018–2019 season, 1.30.

The week with maximal number of weekly incidence ranged from the 4^th^ to 10^th^ week after onset (Table 1). The maximal number of weekly incidence ranged from 32.9 to 64.2 (Table 1).

**Table 1:** Week and number of maximal weekly incidence of influenza patients.

Season	Model	Week	Number
2014–2015	Actual data	5	32.9
	MAS model 1	6 ± 3.7	48.1 ± 25.2
	MAS model 2	7 ± 3.7	43.3 ± 25.6
	MAS model 3	6 ± 3.3	37.7 ± 23.9
	MAS model 4	7 ± 3.6	51.8 ± 15.9
	SIR model	13	40.2
2015–2016	Actual data	4	39.4
	MAS model 1	5 ± 3.0	52.7 ± 33.9
	MAS model 2	6 ± 3.0	56.0 ± 30.9
	MAS model 3	6 ± 2.5	64.6 ± 23.9
	MAS model 4	5 ± 2.2	52.7 ± 17.0
	SIR model	11	46.7
2016–2017	Actual data	10	38.7
	MAS model 1	6 ± 3.5	32.8 ± 19.2
	MAS model 2	7 ± 3.5	29.5 ± 19.3
	MAS model 3	7 ± 3.2	26.7 ± 20.4
	MAS model 4	10 ± 2.2	45.3 ± 11.0
	SIR model	19	16.5
2017–2018	Actual data	9	54.1
	MAS model 1	6 ± 3.6	34.0 ± 20.8
	MAS model 2	7 ± 3.9	31.1 ± 20.0
	MAS model 3	7 ± 4.0	31.4 ± 20.4
	MAS model 4	10 ± 1.7	52.4 ± 15.0
	SIR model	17	21.9
2018–2019	Actual data	7	64.2
	MAS model 1	6 ± 3.7	48.1 ± 25.1
	MAS model 2	7 ± 3.7	43.3 ± 25.5
	MAS model 3	6 ± 3.3	37.1 ± 23.9
	MAS model 4	7 ± 3.5	52.5 ± 16.5
	SIR model	14	39.9

Numbers are reported as mean ± standard deviation or *N*.

MAS, multi-agent system.

### 3.2 The SIR model

The calculation time ranged from 0.808 to 0.112 s (Table 2). The week of the maximal number of weekly incidence ranged from 11 to 19 weeks, and the number of maximal weekly incidence ranged from 16.5 to 46.7 (Table 1). The increase in the weekly incidence was lower, and the week of peak incidence was approximately 8 weeks later than the actual data. The number of maximal weekly incidence was close to the actual data when the peak was before the New Year Holidays (2014–2015 season), and when reduction in incidence was not observed (2015–2016 season). However, when the incidence dramatically increased after the New Year Holidays (2016–2017, 2017–2018, and 2018–2019 seasons), the maximal weekly incidence was around 2 to 3 times higher than the number predicted using the SIR model. The errors between the SIR model and the actual data were larger than the error between the MAS collision models and the actual data (Table 3).

**Table 2:** Calculation time per simulation.

Season	Model	Time (s)
2014–2015	MAS model	1766 ± 70
	SIR model	0.0808
2015–2016	MAS model	1775 ± 84
	SIR model	0.110
2016–2017	MAS model	1745 ± 76
	SIR model	0.0998
2017–2018	MAS model	1739 ± 65
	SIR model	0.104
2018–2019	MAS model	1751 ± 71
	SIR model	0.112

Numbers are reported as mean ± standard deviation. Standard deviation was not available for the SIR model because calculation was performed once.

MAS, multi-agent system.

**Table 3:** Error between the simulation and the actual data.

Season	Model	*N*∗	MAE	RMSE	MAPE
2014–2015	MAS model 1	500	4.00	5.18	55.7%
	MAS model 2	129	3.58	5.09	58.7%
	MAS model 3	43	3.25	4.79	45.3%
	MAS model 4	45	6.23	8.32	94.9%
	SIR model	N/A	15.4	19.3	438%
2015–2016	MAS model 1	500	2.54	3.55	41.9%
	MAS model 2	146	3.87	5.19	73.0%
	MAS model 3	61	5.48	8.34	64.0%
	MAS model 4	46	2.99	3.76	89.7%
	SIR model	N/A	16.9	20.7	921%
2016–2017	MAS model 1	500	9.76	12.1	89.2%
	MAS model 2	97	7.29	10.3	50.5%
	MAS model 3	30	7.14	10.0	49.4%
	MAS model 4	19	3.92	4.52	52.3%
	SIR model	N/A	8.90	12.5	66.6%
2017–2018	MAS model 1	500	9.80	17.0	45.8%
	MAS model 2	99	8.44	15.9	33.7%
	MAS model 3	37	8.29	15.6	38.0%
	MAS model 4	13	3.98	5.83	35.9%
	SIR model	N/A	16.2	21.0	393%
2018–2019	MAS model 1	500	8.41	14.2	118%
	MAS model 2	148	8.85	15.4	122%
	MAS model 3	44	8.46	15.4	113%
	MAS model 4	23	7.49	10.9	132%
	SIR model	N/A	21.9	26.6	925%

MAE, mean absolute error; MAPE, mean absolute percentage error; MAS, multi-agent system; RMSE, root mean squared error.

∗ Number of simulations included in the model.

### 3.3 The MAS collision model: preliminary experiment

Quadratic regression analysis was performed, and a strong positive relationship was observed (*R*^*2*^ = 0.99) between the mean particle velocity and the number of collisions (Figure 2). We assumed that a mean velocity of 0.98 would result in approximately 3,000 collisions per 12 frames including the lower limit of the 95% confidence interval. We also estimated the mean particle velocity to correspond to reduction in collision count to 75% (3,000 × 0.75 = 2,250) during the New Year holidays. Velocity reduction to 70% (0.686) would result in a total collision count of 2,255. Hence, we reduced the particle velocity to 70% during the holidays.

### 3.4 The MAS collision model: main experiment

**Figure 3:**
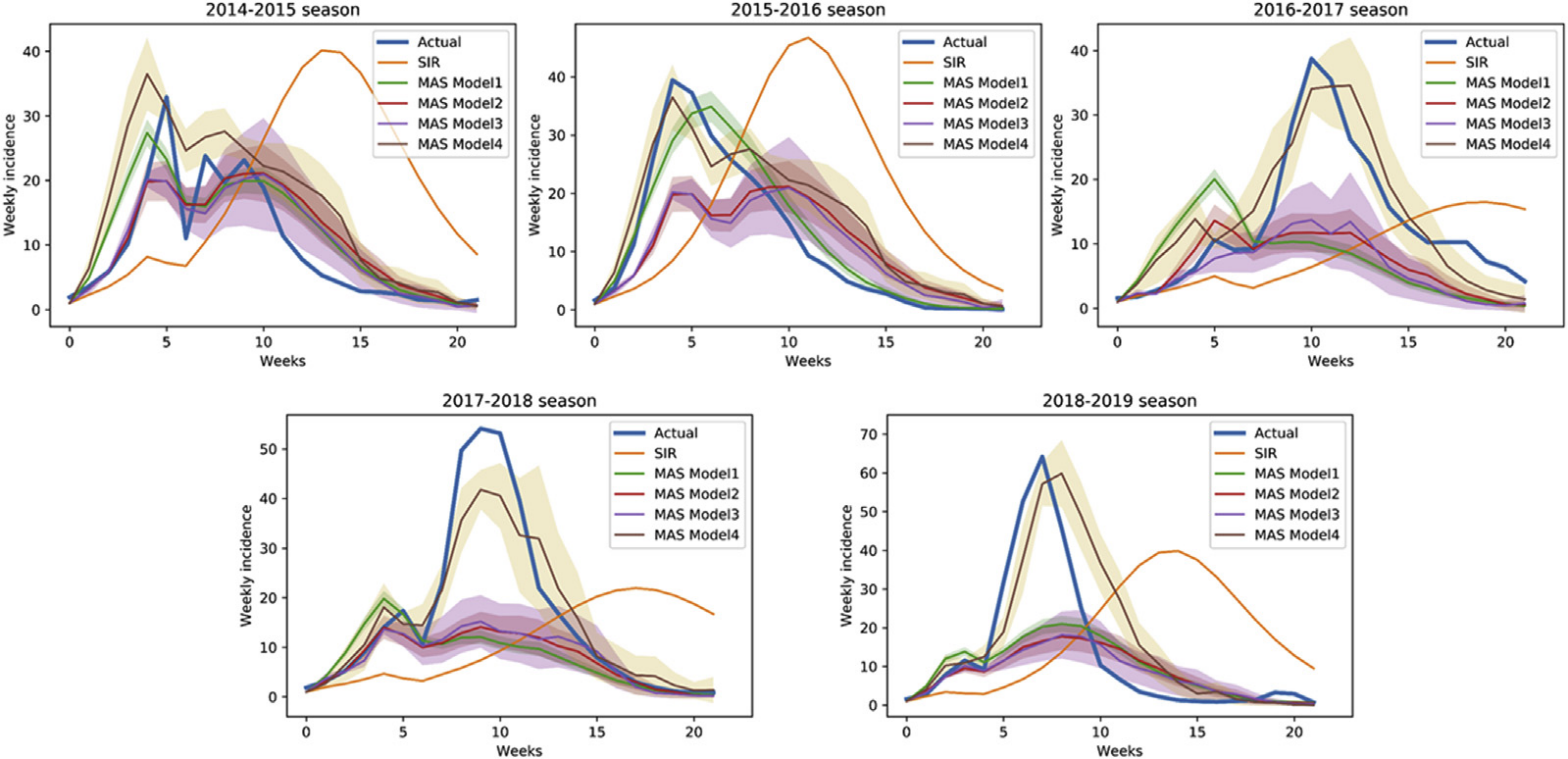
Actual and predicted data of weekly influenza cases in different seasons. Blue and orange lines correspond to the actual incidence data and prediction using the SIR model. Four different models were used for the MAS collision model analysis: Model 1 (green), including all simulations; Model 2 (red), simulations selected by weekly incidence at week 2; Model 3 (purple), simulation selected by weekly incidence at weeks 2 and 4; Model 4 (brown), simulation selected by weekly incidence at the week of peak incidence. Shaded areas represent 95% confidence interval. CI, confidence interval; MAS, multi-agent system.

**Figure 4:**
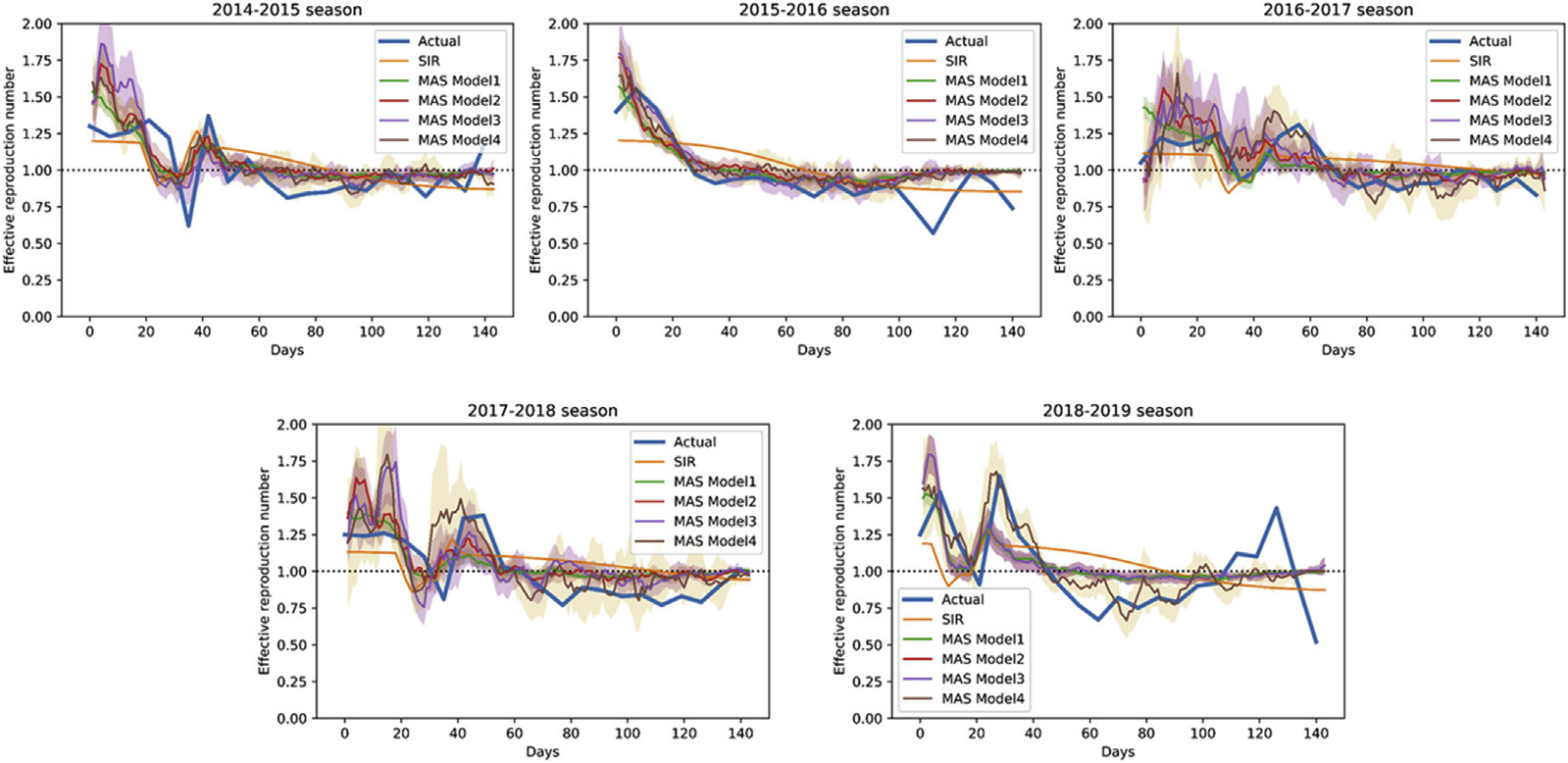
Effective reproduction numbers using actual and predicted data in different seasons. Blue and orange lines correspond to the actual incidence data and prediction using the SIR model. Different models were used for MAS collision model analysis: Model 1 (green), including all simulations; Model 2 (red), simulations selected by weekly incidence at week 2; Model 3 (purple), simulation selected by weekly incidence at weeks 2 and 4; Model 4 (brown), simulation selected by weekly incidence at the week of peak incidence. Shaded areas represent 95% confidence interval. MAS, multi-agent system.

The mean calculation time was approximately 30 min per one simulation (Table 2). The analysis including all simulations (Model 1) corresponded good to the actual data in 2014–2015 and 2015–2016 seasons (Figure 3, Table 3). However, the model failed to predict the high peak incidence after the New Year Holidays in 2016–2017, 2017–2018, and 2018–2019 seasons. In the latter 3 seasons, the peak weekly incidence was higher (Supplementary Figure A) and the week of peak incidence was later (Supplementary Figure B) than expected. The reproduction number after the holidays was higher than the *R*_*0*_, which resulted in a steep curve (Figure 4). Sensitivity analysis showed that the week and number of maximal weekly incidence did not differ between the first and the latter half of the simulations (Supplemental Table 3).

Simulations in models 2 and 3 were selected based on the weekly incidence of week 2, and weeks 2 & 4, respectively. The selected simulations ranged 20–29%, and 6–9% of the total simulations, respectively (Table 3). The prediction error was slightly better in 2014–2015, 2016–2017, 2017–2018 seasons, but it was still difficult to predict the high peak after the New Year Holidays (Figure 3, Supplementary Figure B). The reproduction number after the New Year Holidays was lower than the actual value in the latter 3 seasons (Figure 4).

Model 4 included simulations selected by the week of peak incidence. Therefore, the simulated incidence curve was close to the actual curve in all seasons (Figure 3). The reproduction number was also similar to the actual data (Figure 4). However, the proportion of selected models were smaller than models 2 and 3, ranging from 3% to 9% (Table 3).

## 4 Discussion

The present study applied the MAS collision model to predict the influenza epidemic and tested the method in 5 influenza seasons. A model including all simulated cases (Model 1) predicted the peak number of incidence and week of peak incidence well in 2014–2015 and 2015–2016 seasons, but it underestimated the peak number of weekly incidence in the remaining 2016–2017, 2017–2018, and 2018–2019 seasons. In these three seasons, a small peak was observed before the New Year Holidays, but a second peak after the holidays showed a much higher number of incidence than the first peak. The initial model could not thoroughly predict the second peak, but models which selected the appropriate simulations worked better than the initial model. The proposed method might be close to the pairwise model [23, 24], but this model is different in that pairs are generated visually by the collision of particles. The MAS collision model (especially Model 4) might be criticized that it is just presenting the best fit result, because we filtered the results using the actual incidence at the 4^th^ week, which is the week of the peak incidence in the first two seasons. However, the peak arrived much later (ranging from 7 to 10 weeks) during the last three seasons. The MAS collision model 4 was able to predict the week and intensity of the peak in these seasons.

We mentioned to the model proposed in this study as a MAS collision model, but there are number of studies which used MAS models to predict the transmission of influenza. A simple model just focusing on the number of collisions might not predict the actual curve with good precision. Therefore, a number of studies attempted to improve the precision by including other parameters as follows in order to simulate the daily schedule of each individual: compartments such as home, supermarket, school, and workplaces, temperature, cognition to self-awareness, day of the week, age, and railway line [16, 17]. Sophisticated models including the aforementioned parameters would help to perform a mean field approximation using stochastic models. However, unknown parameters might affect the epidemic curve and the results might change even when the initial conditions are the same. The mean value of the predicted curve might not precisely forecast the actual epidemic each year. Selecting the appropriate simulations after performing numerous simulations using a simplified model would be another approach to increase the precision. The MAS collision model in the present study only focused on the transmission process, hence the calculation time would be shorter than the other sophisticated models. Because of the simplicity of the model, the daily contact number was determined as 3 times per day. This is smaller than the daily contact number in the real world, which ranges from 7 to 18 contacts per day shown in the POLYMOD study [25]. In the model we proposed, the main concept was to focus on the collision, while considering the stochastic nature of each collision. We did not incorporate time schedule or compartment to keep the model simple. In other words, this model is a mean field approximation of the real world. Because the model itself is quite simple, this model could be easily implemented to a compartment model representing each district of Tokyo without increasing the calculation cost too much. Although the contact number per day was low, we adjusted the probability of transmission from an infectious individual to a susceptible individual as R0×γ ∕ 3. This approach might not be robust when the number of individuals included in the model is small, but increasing the number of individuals would make the calculation results robust.

Another strength of the proposed model is that the initial particle velocity was determined to be normally distributed. In the real world, the epidemic curve would substantially change when a “super spreader” is infected. An infected high-velocity-particle would collide more particles than a particle with intermediate velocity. The mean epidemic curve of this model might be close to the SIR model, but the result includes simulations when a highly active infected particle causes a surge in the epidemic curve. Also, the velocity of particles does not need to be normally distributed. If the mean age is young, a Poisson distribution with more particles with higher velocity than a normal distribution could be adopted. The activity of the population could be translated as velocity distribution in a easy way using this method.

The estimated peak using the SIR method in the present study was approximately 8 weeks later than the actual data. This occurred because the *R*_*0*_ derived from the data around the epidemic onset was smaller than the actual reproductive number especially at the beginning of the epidemic. Mercer et al. [26] showed that reproduction numbers are commonly overestimated early in a disease outbreak due to imported cases and outbreaks arising in subpopulations. In the MAS collision model, while some simulations ended with only a few infected individuals, some simulations showed a steep incidence curve. This reflects the rise in transmission rate due to outbreaks in subpopulations. The difference between the SIR model and the MAS collision model is that individuals in each compartment change continuously in the SIR model, but the change is discontinuous in the MAS collision model because a particle could not be divided into small parts. Therefore, a single infected particle might transmit to multiple particles in a short time and make a cluster, which does not arise in the SIR model [24]. The precision of incidence curve using the SIR model could be improved by correcting the reproduction number after the disease outbreak. The strength of the MAS collision model is that prediction could be performed using the reproduction number around the beginning of the season.

A previous study proposed a real-time prediction model of influenza outbreaks by calibrating the parameters used in the SIR model (β and γ) every week [6]. Although the precision of the weekly incidence curve improves with the calibration method, data of peak incidence is necessary to increase the precision, similar to Model 4 in the present study. Therefore, predicting the key parameters using a small data might be difficult. In this context, multi-step prediction method which uses previous annual epidemic data could more accurately predict the epidemic [27]. A previous study using this method showed that implementing multiple single-output prediction in a six-layer long short-term memory structure achieved good accuracy to predict influenza incidence of 2–13 weeks ahead. A study by Yang et al. [28] attempted to forecast influenza epidemics in Hong Kong using Kalman filter in conjunction with the SIR model. In this model, when a new observation arrives, the system (including all model variables and parameters) is updated per filter algorithm. Although we acknowledge that this filtering is a sophisticated method, the main parameters which is used to calculate the SIR model is constantly renewed. However, the principal finding of the present study is that the incidence curve with good correlation with the actual data could be selected without changing the *R*_*0*_ value. This is explained by the stochastic nature of the MAS collision model.

Numerous factors influence the transmission of influenza including vaccination rate [29], age [30], and temperature [1]. Also, ascertainability might change with different age groups [30]. These factors were not included in the MAS collision model. Influenza epidemic curve is influence by these factors, and including these factors might further help to increase the precision. McGowan et al [31] showed the superiority of statistical models with stochasticity vs. SIR models and concluded that SIR models should include major environmental determinants for predicting peaks. Additionally, ensemble forecasts including various prediction models perform better than one single model and the SIR model can also be included into the ensemble. One challenge in the weekly incidence data of Tokyo is how to account for the decrease in detected patients during the New Year Holidays. First, the number of patients who visit to hospitals and clinics decreases because a lot of them are closed during the holidays. Second, the population of Tokyo decreases around 50–60% during the holidays because many residents return to their hometown (https://www.blogwatcher.co.jp/case/report_newyear_2017/). If the reproductive number maintains the same value, the weekly incidence in the first week of the new year should increase dramatically. The actual data shows that the incidence curve shifts towards right without showing a severe increase after the holidays. Therefore, we determined that the transmission declines during the holidays, but further investigation is needed to confirm the actual reduction in the reproduction number. Using a real-time surveillance data might serve to answer this question [32, 33].

We acknowledge the following limitations in this study. First, the proposed model was verified in only 5 influenza seasons in Tokyo. Further study is needed to validate the MAS collision method in different epidemic seasons and different cities or countries. Second, there are multiple methods to estimate the *R*_*0*_ value [3, 34] and the results might change when different methods are used. Third, we did not consider the vaccination rate in Japan. The vaccination rate is gradually increasing from 14.9% to 28.0% between 2014 and 2018 (https://www.mhlw.go.jp/shingi/2008/06/dl/s0618-9a.pdf). The increase in the vaccination rate might have reduced the transmission of influenza. Vaccine effectiveness of influenza differs between seasons [35]. Forth, the model we proposed was based on the SIR model, but the precision might improve using other models such as the SEIR model. The latent period of influenza is about 2 days and the transmission occurs 1–2 days before onset [21]. The infectious period starts just after the exposure of influenza. Therefore, the SIR model adopted in this study would fit well for influenza virus transmission. Finally, the calculation time was 30 min per simulation, which could be reduced by advancement in CPU or improvement in programming.

## 5 Conclusions

We applied the MAS collision model to predict seasonal influenza epidemic for 5 years in Tokyo. The model predicted the epidemic curve with good accuracy by selecting the simulations using the actual data without changing the initial parameters such as the basic reproduction number and infection time.

## Declarations

### Author contribution statement

Nobuo Tomizawa: Conceived and designed the experiments; Performed the experiments; Analyzed and interpreted the data; Contributed reagents, materials, analysis tools or data; Wrote the paper.

Kanako K Kumamaru: Analyzed and interpreted the data; Wrote the paper.

Koh Okamoto: Conceived and designed the experiments; Analyzed and interpreted the data; Wrote the paper.

Shigeki Aoki: Conceived and designed the experiments; Wrote the paper.

### Funding statement

This work was supported by Research Funds of the 10.13039/501100003478Ministry of Health, Labour and Welfare (20IA1012).

### Data availability statement

Data included in article/supplementary material/referenced in article.

### Declaration of interests statement

The authors declare no conflict of interest.

### Additional information

No additional information is available for this paper.

## Figures and Tables

**Suppl_FigureA:**
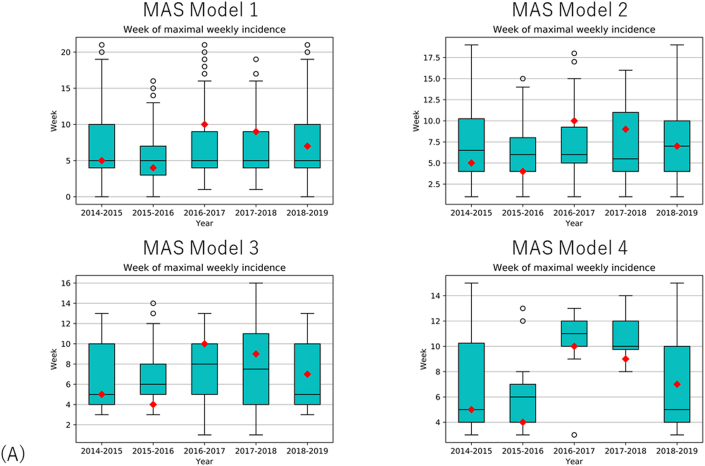


**Suppl_FigureB:**
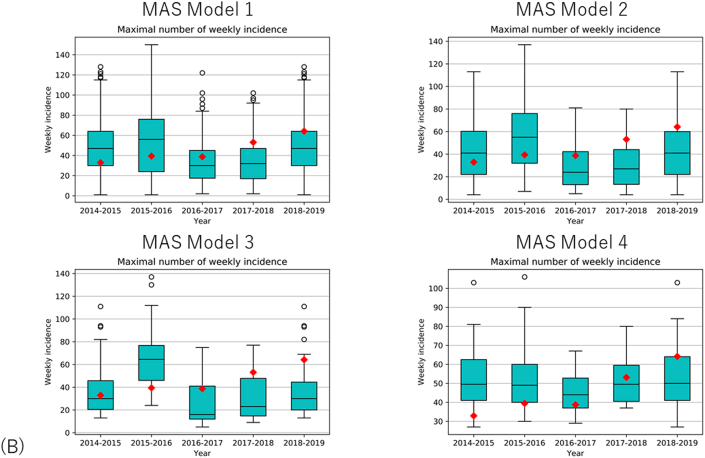


## Appendix A Supplementary data

Collision_Flu_Heliyon_spl Collision_Flu_Heliyon_spl
