# Supplementary material for: Multi-agent system collision model to predict the transmission of seasonal influenza in Tokyo from 2014–2015 to 2018–2019 seasons
Source: Heliyon. 2021 Aug 23;7(8):e07859. doi: 10.1016/j.heliyon.2021.e07859 (PMC8391024; doi:10.1016/j.heliyon.2021.e07859)
Supplement: Collision_Flu_Heliyon_spl [file mmc1.docx]

**Multi-agent system collision model to predict the transmission of seasonal influenza in Tokyo from 2014-2015 to 2018-2019 seasons**

Nobuo Tomizawa, MD, PhD,^a^ Kanako K Kumamaru, MD, PhD,^a^ Koh Okamoto, MD, PhD,^b^ Shigeki Aoki, MD, PhD,^a^

^a^Department of Radiology, Juntendo University Graduate School of Medicine, Tokyo, Japan

^b^Department of Infectious Diseases, The University of Tokyo Hospital, Tokyo, Japan

**Supplemental Table 1** Data of influenza incidence in Tokyo

| Day | 2014-2015 | 2015-2016 | 2016-2017 | 2017-2018 | 2018-2019 |
| --- | --- | --- | --- | --- | --- |
| 0 | 1.92 | 1.67 | 1.57 | 1.86 | 1.57 |
| 7 | 3.62 | 3.79 | 1.75 | 3.17 | 2.68 |
| 14 | 5.86 | 11.17 | 2.79 | 5.26 | 7.79 |
| 21 | 10.15 | 25.98 | 4.02 | 9.03 | 11.53 |
| 28 | 20.6 | 39.43 | 6.24 | 14.03 | 9.2 |
| 35 | 32.9 | 37.25 | 10.58 | 17.37 | 31.71 |
| 42 | 11.04 | 29.89 | 9.06 | 10.78 | 52.65 |
| 49 | 23.8 | 25.82 | 9.22 | 22.84 | 64.18 |
| 56 | 19.71 | 22.86 | 15.17 | 49.67 | 45.71 |
| 63 | 23.14 | 19.44 | 28.58 | 54.1 | 25.17 |
| 70 | 18.88 | 14.86 | 38.73 | 53.13 | 10.32 |
| 77 | 11.43 | 9.31 | 35.49 | 39.56 | 6.62 |
| 84 | 7.74 | 7.46 | 26.18 | 21.96 | 3.47 |
| 91 | 5.33 | 4.9 | 22.35 | 16.82 | 2.2 |
| 98 | 4.07 | 3.6 | 15.65 | 12.05 | 1.27 |
| 105 | 2.84 | 2.8 | 12.47 | 7.9 | 0.99 |
| 112 | 2.7 | 1.4 | 10.19 | 5.28 | 0.83 |
| 119 | 2.39 | 0.4 | 10.23 | 2.92 | 1.09 |
| 126 | 1.53 | 0.25 | 10.23 | 1.9 | 1.36 |
| 133 | 1.4 | 0.25 | 7.27 | 1.1 | 3.26 |
| 140 | 1 | 0.2 | 6.32 | 0.87 | 2.9 |
| 147 | 1.5 | 0.1 | 4.2 | 0.9 | 0.69 |

**Supplemental Table 2** Relationship of reproduction number and weekly incidence ratio

| *R* | Ratio* | *R* | Ratio* | *R* | Ratio* | *R* | Ratio* | *R* | Ratio* |
| --- | --- | --- | --- | --- | --- | --- | --- | --- | --- |
| 0.30 | 0.081 | 0.64 | 0.370 | 0.98 | 0.954 | 1.32 | 1.952 | 1.66 | 3.516 |
| 0.31 | 0.086 | 0.65 | 0.382 | 0.99 | 0.977 | 1.33 | 1.990 | 1.67 | 3.572 |
| 0.32 | 0.091 | 0.66 | 0.395 | 1.00 | 1.000 | 1.34 | 2.027 | 1.68 | 3.629 |
| 0.33 | 0.097 | 0.67 | 0.408 | 1.01 | 1.024 | 1.35 | 2.065 | 1.69 | 3.687 |
| 0.34 | 0.103 | 0.68 | 0.421 | 1.02 | 1.047 | 1.36 | 2.104 | 1.70 | 3.745 |
| 0.35 | 0.109 | 0.69 | 0.435 | 1.03 | 1.072 | 1.37 | 2.143 | 1.71 | 3.804 |
| 0.36 | 0.115 | 0.70 | 0.449 | 1.04 | 1.096 | 1.38 | 2.182 | 1.72 | 3.864 |
| 0.37 | 0.121 | 0.71 | 0.463 | 1.05 | 1.121 | 1.39 | 2.223 | 1.73 | 3.925 |
| 0.38 | 0.128 | 0.72 | 0.477 | 1.06 | 1.147 | 1.40 | 2.263 | 1.74 | 3.986 |
| 0.39 | 0.134 | 0.73 | 0.491 | 1.07 | 1.172 | 1.41 | 2.304 | 1.75 | 4.047 |
| 0.40 | 0.141 | 0.74 | 0.506 | 1.08 | 1.199 | 1.42 | 2.346 | 1.76 | 4.110 |
| 0.41 | 0.148 | 0.75 | 0.521 | 1.09 | 1.225 | 1.43 | 2.388 | 1.77 | 4.173 |
| 0.42 | 0.155 | 0.76 | 0.537 | 1.10 | 1.252 | 1.44 | 2.431 | 1.78 | 4.237 |
| 0.43 | 0.163 | 0.77 | 0.553 | 1.11 | 1.279 | 1.45 | 2.474 | 1.79 | 4.301 |
| 0.44 | 0.171 | 0.78 | 0.569 | 1.12 | 1.307 | 1.46 | 2.518 | 1.80 | 4.367 |
| 0.45 | 0.178 | 0.79 | 0.585 | 1.13 | 1.335 | 1.47 | 2.562 | 1.81 | 4.433 |
| 0.46 | 0.187 | 0.80 | 0.601 | 1.14 | 1.364 | 1.48 | 2.607 | 1.82 | 4.499 |
| 0.47 | 0.195 | 0.81 | 0.618 | 1.15 | 1.393 | 1.49 | 2.653 | 1.83 | 4.567 |
| 0.48 | 0.203 | 0.82 | 0.636 | 1.16 | 1.422 | 1.50 | 2.699 | 1.84 | 4.635 |
| 0.49 | 0.212 | 0.83 | 0.653 | 1.17 | 1.452 | 1.51 | 2.745 | 1.85 | 4.704 |
| 0.50 | 0.221 | 0.84 | 0.671 | 1.18 | 1.482 | 1.52 | 2.793 | 1.86 | 4.774 |
| 0.51 | 0.230 | 0.85 | 0.689 | 1.19 | 1.513 | 1.53 | 2.840 | 1.87 | 4.844 |
| 0.52 | 0.239 | 0.86 | 0.707 | 1.20 | 1.544 | 1.54 | 2.889 | 1.88 | 4.915 |
| 0.53 | 0.249 | 0.87 | 0.726 | 1.21 | 1.576 | 1.55 | 2.938 | 1.89 | 4.987 |
| 0.54 | 0.259 | 0.88 | 0.745 | 1.22 | 1.608 | 1.56 | 2.987 | 1.90 | 5.060 |
| 0.55 | 0.269 | 0.89 | 0.765 | 1.23 | 1.640 | 1.57 | 3.037 | 1.91 | 5.134 |
| 0.56 | 0.279 | 0.90 | 0.784 | 1.24 | 1.673 | 1.58 | 3.088 | 1.92 | 5.208 |
| 0.57 | 0.290 | 0.91 | 0.804 | 1.25 | 1.706 | 1.59 | 3.139 | 1.93 | 5.283 |
| 0.58 | 0.300 | 0.92 | 0.825 | 1.26 | 1.740 | 1.60 | 3.191 | 1.94 | 5.359 |
| 0.59 | 0.311 | 0.93 | 0.845 | 1.27 | 1.774 | 1.61 | 3.244 | 1.95 | 5.436 |
| 0.60 | 0.323 | 0.94 | 0.866 | 1.28 | 1.809 | 1.62 | 3.297 | 1.96 | 5.514 |
| 0.61 | 0.334 | 0.95 | 0.888 | 1.29 | 1.844 | 1.63 | 3.351 | 1.97 | 5.592 |
| 0.62 | 0.346 | 0.96 | 0.910 | 1.30 | 1.880 | 1.64 | 3.405 | 1.98 | 5.671 |
| 0.63 | 0.358 | 0.97 | 0.932 | 1.31 | 1.916 | 1.65 | 3.460 | 1.99 | 5.752 |

* Ratio means (weekly incidence at week *N*+1) ∕ (weekly incidence at week *N*).

*R*, reproduction number

| *R* | Ratio* | *R* | Ratio* |
| --- | --- | --- | --- |
| 2.00 | 5.832 | 2.34 | 9.132 |
| 2.01 | 5.914 | 2.35 | 9.247 |
| 2.02 | 5.997 | 2.36 | 9.362 |
| 2.03 | 6.080 | 2.37 | 9.479 |
| 2.04 | 6.165 | 2.38 | 9.597 |
| 2.05 | 6.250 | 2.39 | 9.716 |
| 2.06 | 6.336 | 2.40 | 9.837 |
| 2.07 | 6.423 | 2.41 | 9.958 |
| 2.08 | 6.511 | 2.42 | 10.080 |
| 2.09 | 6.600 | 2.43 | 10.204 |
| 2.10 | 6.689 | 2.44 | 10.329 |
| 2.11 | 6.780 | 2.45 | 10.455 |
| 2.12 | 6.871 | 2.46 | 10.582 |
| 2.13 | 6.964 | 2.47 | 10.710 |
| 2.14 | 7.057 | 2.48 | 10.840 |
| 2.15 | 7.152 | 2.49 | 10.971 |
| 2.16 | 7.247 | 2.50 | 11.103 |
| 2.17 | 7.343 |  |  |
| 2.18 | 7.440 |  |  |
| 2.19 | 7.538 |  |  |
| 2.20 | 7.638 |  |  |
| 2.21 | 7.738 |  |  |
| 2.22 | 7.839 |  |  |
| 2.23 | 7.941 |  |  |
| 2.24 | 8.044 |  |  |
| 2.25 | 8.148 |  |  |
| 2.26 | 8.253 |  |  |
| 2.27 | 8.359 |  |  |
| 2.28 | 8.467 |  |  |
| 2.29 | 8.575 |  |  |
| 2.30 | 8.684 |  |  |
| 2.31 | 8.795 |  |  |
| 2.32 | 8.906 |  |  |
| 2.33 | 9.018 |  |  |

**Supplemental Table 3** Comparison of week and number of maximal weekly incidence of influenza patients between simulations 1-250 and 251-500

| Season | Model | Week | Number |
| --- | --- | --- | --- |
| 2014- | Entire simulations | 6 ± 3.7 | 48.1 ± 25.2 |
| 2015 | Simulations 1-250 | 7 ± 4.0 | 47.5 ± 24.2 |
|  | Simulations 251-500 | 6 ± 3.6 | 48.5 ± 26.1 |
| 2015- | Entire simulations | 5 ± 3.0 | 52.7 ± 33.9 |
| 2016 | Simulations 1-250 | 5 ± 3.0 | 54.2 ± 34.0 |
|  | Simulations 251-500 | 5 ± 3.0 | 51.0 ± 33.6 |
| 2016- | Entire simulations | 6 ± 3.5 | 32.8 ± 19.2 |
| 2017 | Simulations 1-250 | 6 ± 3.5 | 33.1 ± 18.9 |
|  | Simulations 251-500 | 6 ± 3.6 | 32.5 ± 19.6 |
| 2017- | Entire simulations | 6 ± 3.6 | 34.0 ± 20.8 |
| 2018 | Simulations 1-250 | 6 ± 3.3 | 34.2 ± 21.3 |
|  | Simulations 251-500 | 6 ± 3.9 | 33.8 ± 20.3 |
| 2018- | Entire simulations | 6 ± 3.7 | 48.1 ± 25.1 |
| 2019 | Simulations 1-250 | 7 ± 4.0 | 47.6 ± 24.1 |
|  | Simulations 251-500 | 6 ± 3.6 | 48.6 ± 26.0 |

Numbers are reported as mean ± standard deviation or *N*.

**Supplemental Figure** Box and whisker plot of maximal number of weekly incidence (A) and week of maximal weekly incidence (B) in different seasons. Different models were used for MAS collision model analysis: Model 1, including all simulations; Model 2, simulations selected by weekly incidence at week 2; Model 3, simulation selected by weekly incidence at weeks 2 and 4; Model 4, simulation selected by weekly incidence at the week of peak incidence.

Note: Box, 1^st^-3^rd^ quartiles; bold line, median; whiskers, minimum and maximum values; circle, outlier; red square, actual value.

MAS, multi-agent system
